# miR135a administration ameliorates brain ischemic damage by preventing TRPM7 activation during brain ischemia

**DOI:** 10.1111/cns.14448

**Published:** 2023-09-18

**Authors:** P. Cepparulo, P. Brancaccio, R. Sirabella, S. Anzilotti, N. Guida, G. Laudati, V. Valsecchi, A. Vinciguerra, V. Viscardi, L. D'Esposito, L. Formisano, L. Annunziato, G. Pignataro, O. Cuomo

**Affiliations:** ^1^ Division of Pharmacology, Department of Neuroscience, School of Medicine University of Naples Federico II Naples Italy; ^2^ Department of Science and Technology University of Sannio Benevento Italy; ^3^ Department of Biomedical Sciences and Public Health University “Politecnica delle Marche” Ancona Italy; ^4^ Veterinary Service Center University of Naples Federico II Naples Italy; ^5^ IRCCS SYNLAB SDN S.p.A. Naples Italy

**Keywords:** cerebral ischemia, ionic homeostasis, miR135, neuroprotection, TRPM7

## Abstract

**Background:**

miRNA‐based strategies have recently emerged as a promising therapeutic approach in several neurodegenerative diseases. Unregulated cation influx is implicated in several cellular mechanisms underlying neural cell death during ischemia. The brain constitutively active isoform of transient receptor potential melastatin 7 (TRPM7) represents a glutamate excitotoxicity‐independent pathway that significantly contributes to the pathological Ca^2+^ overload during ischemia.

**Aims:**

In the light of these premises, inhibition of TRPM7 may be a reasonable strategy to reduce ischemic injury. Since TRPM7 is a putative target of miRNA135a, the aim of the present paper was to evaluate the role played by miRNA135a in cerebral ischemia. Therefore, the specific objectives of the present paper were: (1) to evaluate miR135a expression in temporoparietal cortex of ischemic rats; (2) to investigate the effect of the intracerebroventricular (icv) infusion of miR135a on ischemic damage and neurological functions; and (3) to verify whether miR135a effects may be mediated by an alteration of TRPM7 expression.

**Methods:**

miR135a expression was evaluated by RT‐ PCR and FISH assay in temporoparietal cortex of ischemic rats. Ischemic volume and neurological functions were determined in rats subjected to transient middle cerebral artery occlusion (tMCAo) after miR135a intracerebroventricular perfusion. Target analysis was performed by Western blot.

**Results:**

Our results demonstrated that, in brain cortex, 72 h after ischemia, miR135a expression increased, while TRPM7 expression was parallelly downregulated. Interestingly, miR135a icv perfusion strongly ameliorated the ischemic damage and improved neurological functions, and downregulated TRPM7 protein levels.

**Conclusions:**

The early prevention of TRPM7 activation is protective during brain ischemia.

## Graphical abstract

The present paper showed that icv administration of miR135a ameliorates brain ischemia and improves general and focal neurological deficits by mediating an early reduction in TRPM7 levels in the cortex. This finding supports the idea that an early downregulation of TRPM7 expression may help neurons to survive the ischemic injury.

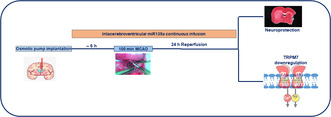

## 1 INTRODUCTION

MicroRNAs are endogenous noncoding RNAs that serve as important regulators of gene expression. Interestingly, miRNA‐based strategies, using miRNA mimics or antagomirs, have recently emerged as a promising therapeutic approach in several neurodegenerative diseases.^1^

It is well established that a perturbation of cellular homeostasis of monovalent or divalent cation influx is implicated in several different cellular mechanisms, such as excitotoxicity, apoptosis, and oxidative stress, underlying neural cell death during ischemia. Interestingly, transient receptor potential channels, also termed TRP channels, are a class of non‐selective cation channels located on the cell membrane of various cell types.^2^, ^3^ In mammals, TRPs are thought to mediate capacitive calcium entry into the cell. Different TRP channels are activated by different physical and chemical stimuli. The diverse gating mechanisms of TRP channels make them good cellular signal integrators critical for physiological and pathological functions.

In line with properties and physiological functions of the brain constitutively active isoform of transient receptor potential melastatin 7 (TRPM7), activation of TRPM7 during ischemic conditions represents a relevant glutamate excitotoxicity‐independent pathway that significantly contributes to the pathological Ca^2+^ overload.

Indeed, TRPM7 is able to sense the extracellular concentration of divalent ions and to maintain intracellular Mg^2+^ homeostasis during episodes that lead to intense neuronal activity, such as ischemia.^4^, ^5^ In addition, a decrease in extracellular pH strongly potentiates the activity of TRPM7 channel, suggesting its role in acidic pathophysiological conditions.

In the light of these premises, finding a way to reduce TRPM7 activation during ischemia may be a reasonable strategy to reduce ischemic injury. Considering the *lack* of pharmacological inhibitors of TRPM7 and taking into account recent evidence that TRPM7 may be considered a putative target of miRNA135a,^6^ the aims of the present paper were to evaluate the effect of the miRNA135a in cerebral ischemia and to verify whether TRPM7 may be responsible for miR135a effects during ischemic conditions.

Therefore, the specific objectives of the present work were as follows: (1) to evaluate miR135a expression in temporoparietal cortex of ischemic rats; (2) to investigate the effect of the icv infusion of miR135a on ischemic damage and neurological functions; and (3) to verify whether miR135a effects on brain ischemia development may be mediated by an alteration of TRPM7 expression.

## 2 MATERIALS AND METHODS

### 2.1 Chemicals

miR135a mimic (mmu‐miR‐135a miRCURY LNA miRNA, ID: YM00470912) and the corresponding negative control (negative control miRCURY LNA miRNA mimic, ID: YM00479902) were purchased from QIAGEN.

### 2.2 Rat primary cortical neurons

Rat primary cortical neurons were prepared from 17‐day‐old Wistar rat embryos (Charles River).^7^ Briefly, rats were first anesthetized and then decapitated to minimize animal pain and distress. Dissection and dissociation were performed in Ca^2+^/Mg^2+^‐free phosphate‐buffered saline (PBS) containing glucose (30 mM). Tissues were incubated with papain for 10 min at 37°C and dissociated by trituration in Earle's Balanced Salt Solution (EBSS) containing DNAse (0.16 U/mL), bovine serum albumin (10 mg/mL), and ovomucoid (10 mg/mL). Neurons were plated in plastic Petri dishes (Falcon™ Becton‐Dickinson) pre‐coated with poly‐D‐lysine (20 μg/mL) and were grown in MEM/F12 (Life Technologies) containing glucose, 5% of deactivated fetal bovine serum (FBS), and 5% of horse serum (HS, Life Technologies), glutamine (2 mM/L), penicillin (50 Units/mL), and streptomycin (50 μg/mL) (Invitrogen). Within 48 h of plating, cytosine arabinoside (arabinoside‐C) (10 μM) was added to prevent non‐neuronal cell growth. Neurons were cultured at 37°C in a humidified 5% CO_2_ atmosphere and used after 7–10 days of culture. Cell density was 5 × 10^6^ cells/well of 60 mm for analysis of qRT‐PCR and 15 × 10^6^ cells/well of 100 mm for Western blot analysis.

### 2.3 miR135a mimic and antagomir transfection in rat cortical neurons

Primary rat cortical neurons were transfected with 50 nM miR‐135a mimic or miR‐135a anti‐miRNA and respective Negative Controls. HiPerFect Transfection Reagent was used as transfection agent, according to the manufacturer's protocol. After an incubation period of 24 h, the medium was replaced and cells were harvested and used for Western blot or PCR analysis.^8^

### 2.4 miR135a expression by RT‐PCR analysis

Total RNA from temporoparietal cortex and neurons was extracted following supplier's instructions (Life Technologies), and cDNA was synthesized using 0.5 or 2 mg of total RNA to obtain miRNA‐specific cDNA or total cDNA, respectively, with the high‐capacity transcription kit following supplier's instruction (Life Technologies). Quantitative real‐time PCR was performed with TaqMan assays in a 7500 real‐time PCR system (Life Technologies). Changes in miRNA levels were determined as the difference in threshold cycle (2^−ΔΔCt^) between miR135a (ID: 000460) and the miRNA control assay 4.5S RNA (ID: 001716).^9^

### 2.5 TRPM7 expression by Western blot analysis

Samples from rat temporoparietal cortex or neurons were homogenized in a lysis buffer (50 mmol/L Tris–HCl [pH 7.5], 100 mmol/L NaCl, 1% Triton X‐100) containing protease and phosphatase inhibitors. After centrifugation at 12,000 × *g* at 4°C for 20 min, the supernatants were collected. Protein concentration was estimated using the Bradford method (Bio‐Rad Laboratories) by means of a spectrophotometer (Eppendorf). Then, 80–100 μg of protein lysate was mixed with a Laemmli sample buffer and boiled at 95°C for 5 min. The samples were resolved by SDS‐PAGE at 8% and transferred to nitrocellulose membranes Amersham TM Hybond TM‐ECL (GE Healthcare). Non‐specific sites were blocked with 5% non‐fat milk incubation in Tris‐buffered saline (TBS) solution with 0.1% Tween 20, for 1 h at room temperature. Blots were probed with antibodies for TRPM7 (1:750; Abcam), β‐actin (1:10,000, Sigma‐Aldrich), diluted in 5% non‐fat milk, overnight at 4°C. Then, they were detected using horseradish peroxidase‐conjugated secondary antibody (1:2000, goat and mouse; Amersham Pharmacia Biotech Italia) for 60 min at room temperature in 5% non‐fat milk and an enhanced luminescence kit (Amersham Pharmacia Biotech).^9^

### 2.6 miR135a expression by fluorescence in situ hybridization (FISH)

For in situ hybridization, all procedures were performed in autoclaved solutions and RNAse‐free conditions. Rats were perfused with 1× PBS and 4% paraformaldehyde solution in PBS. The brain tissues from ischemic and control rats were fixed in 4% paraformaldehyde solution in PBS overnight at 4°C and subsequently cryoprotected in 30% sucrose in PBS overnight at 4°C and cryosectioned at 10‐μm thickness. Frozen tissue sections were prepared following the description of miRNA protocol for in situ hybridization on frozen sections (Exiqon).

Briefly, brain sections from ischemic and control animals 72 h after reperfusion were submerged in neutral buffered formalin (10%) for 15 min and then washed in PBS three times for 5 min. Sections were incubated in proteinase K buffer containing 1 M Tris–HCl (pH 7.4), 0.5 M EDTA, 5 M NaCl, and proteinase K (15 μg/mL) in RNase‐free water for 10 min at 37°C, and then, the sections were washed three times for 3 min in PBS. Sections were then incubated in 3% H_2_O_2_ for 5 min to inhibit endogenous peroxidase activity and then washed in PBS three times for 3 min. Sections were sequentially hybridized for 1 h at 55° for mir‐135a‐5p (5′DIG and 3′DIG). The final concentration of the probe was 20 nM. The sections were hybridized in hybridization buffer containing 50% deionized formamide, 0.3 M NaCl, 20 mM Tris–HCl (pH 8.0), 5 mM EDTA, 10 mM NaPO4 (pH 8.0), 10% dextran sulfate, 1× Denhardt's solution, 0.5 mg/mL yeast RNA, and probe. Post‐hybridization washes were performed sequentially twice for 5 min at hybridization temperature in 5× saline sodium citrate (SSC) buffer, three times for 5 min at hybridization temperature in 1× SSC buffer, twice for 5 min at hybridization temperature in 0.2× SSC, and once for 5 min at room temperature in 0.2× SSC buffer. Following the stringent washing, the sections were incubated in blocking solution containing 2% sheep serum and 1% BSA in PBS with 0.1% Tween 20 for 15 min at room temperature. Then, the sections were incubated for 60 min with anti‐digoxigenin‐peroxidase (POD), antigen‐binding fragments (Fabs; Roche Diagnostics) diluted 1:400 in 1% sheep serum, 1% BSA, and PBS with 0.05% Tween 20×.^9^ Then, the sections were washed in PBS three times for 5 min and incubated for 5 min in Cy2‐conjugated tyramide (tyramide signal amplification [TSA] Plus Fluorescein kit, PerkinElmer) by diluting TSA stock solution 1:50 in 1× amplification diluent. After washing three times for 10 min with TBS, sections were incubated for 30 min in 3% H_2_O_2_ in TBS to quench peroxidase activity from the initial TSA reaction. After washing, sections were incubated with the following primary antibodies in blocking solution: anti‐NeuN 1:200 (Millipore) overnight. Sections were sequentially washed three times for 10 min with PBS and then incubated for 2 h in Alexa Fluor 594‐conjugated donkey anti‐mouse/rabbit antiserum diluted 1:300 in blocking solution. Following washing three times for 10 min with PBS, sections were incubated with Hoechst for 40 min and mounted onto slides using Fluoromount aqueous mounting medium (Sigma), air‐dried, and stored in a dark room. As controls, the sections were incubated without the anti‐digoxigenin‐POD or without the TSA Plus Fluorescein kit or primary antibodies, and the immunoreactivity was completely abolished (data not shown).^9^ To obtain an indirect measure of the amount of miR‐135a‐5p in neurons, image analysis of NeuN was performed by NIH image software by measuring the intensity of fluorescent miR‐135a‐5p immunolabeling in 120 NeuN‐positive neurons for each group. The intensity of miR‐135a‐5p immunoreactivity was expressed in arbitrary units. The colocalization was performed by counting the number of NeuN/mir135 positive cells and normalized to the total number of Hoechst positive cells per slide. Digital images were taken with a ×40 objective, and identical laser power settings and exposure times were applied to all images from each experimental set. Images from the same areas of each brain region were compared.^10^, ^11^

### 2.7 In vivo experimental groups

Seventy‐two Sprague Dawley male rats (Charles River Laboratories) weighing 150–170 g were housed under diurnal lighting conditions (12‐h light/12‐h dark). It has been calculated that about 20% of the animals used were excluded from the experimental groups due to the absence of ischemic lesions or to mortality related to the experimental procedure.

Experiments were performed according to international guidelines for animal research. The experimental protocol was approved by the Animal Care Committee of the “Federico II” University of Naples.

### 2.8 Transient focal ischemia

Transient focal ischemia was induced by suture occlusion of the middle cerebral artery (MCA) in male rats anesthetized using a mixture of oxygen and sevoflurane at 3.5% (medical oxygen concentrator LFY‐I‐5A). A 5‐0 surgical monofilament nylon suture (Doccol) was inserted from the right external carotid artery into the right internal carotid artery and advanced into the circle of Willis up to the branching point of the MCA, thereby occluding the MCA.^11^ The filament was left in place for 100‐min. Achievement of ischemia was confirmed by monitoring regional cerebral blood flow in the area of the right MCA. Cerebral blood flow was monitored through a disposable microtip fiber optic probe (diameter, 0.5 mm) connected through a master probe to a laser Doppler computerized main unit (PF5001; Perimed) and analyzed using PSW Perisoft 2.5. Animals not showing a cerebral blood flow reduction of at least 70% were excluded from the experimental group, as were animals that died after ischemia induction. Rectal temperature was maintained at 37 ± 0.5°C with a thermostatically controlled heating pad and lamp. All surgical procedures were performed under an operating stereomicroscope. Transient middle cerebral artery occlusion (tMCAO) was induced, as previously described, by individuals who did not implant osmotic pumps in animals.^12^

For the evaluation of TRPM7 and miR135a expression, rats were sacrificed 12–24–72 h after ischemia induction; for the evaluation of ischemic volume, rats were sacrificed 24 h after ischemia induction.

### 2.9 *miR135a* mimic icv administration

The continuous release of miR135a mimic into the brain lateral ventricle was achieved by using osmotic pumps (Alzeth). The osmotic pumps were prefilled with miR135a mimic or corresponding negative control in a blinded manner by individuals who did not perform tMCAO surgery on animals. Implantation of the osmotic pump frame was carried out in rats positioned on a stereotaxic apparatus 6 h before the induction of transient ischemia. The osmotic pump was connected to a brain infusion kit (Alzeth, no. 0004760) made of a stainless steel cannula that was implanted into the right lateral ventricle using the stereotaxic coordinates from the bregma: 0.4 mm caudal, 2 mm lateral, and 2 mm below the dura and secured to the skull with dental cement. The pump was placed in the skin fold on the neck of the rat. miR135a mimic and the corresponding negative control were diluted to the final concentration in a previously filtered saline solution (0.9% NaCl g/L). The miRNA mimic was icv administered at the concentration 8 μmol/L (9 μg/kg body weight), and the release of miRNA mimic by the osmotic pump within the rat cerebral ventricle was set up at a speed of 1 μL/h. In order to verify the possible protective effect of miRNA135a administered after tMCAO, a subgroup of rats received a single icv miR135a mimic administration 3 h after ischemia onset. In this subgroup of animal, the amount of mimic administered was the same as the one released from the osmotic pumps during the continuous perfusion (1 nmol/kg). Rectal temperature was maintained at 37 ± 0.5°C with a thermostatically controlled heating pad during the whole surgical procedure. In the animals, a catheter was inserted into the femoral artery to measure arterial blood gasses before and after ischemia (Rapid Laboratory 860; Chiron Diagnostics). All surgical procedures were performed under an operating stereomicroscope in a blinded manner.^12^

### 2.10 Evaluation of the infarct volume

For the evaluation of ischemic volume, animals were killed through sevoflurane overdose 24 h after ischemia. Brains were quickly removed, sectioned coronally at 1‐mm intervals, and stained by immersion in the vital dye (2% in PBS) 2,3,5‐triphenyltetrazolium chloride (TTC) (Figure 5). The infarct volume was calculated by summing the infarction areas of each section and by multiplying the total by slice thickness (1 mm) through image analysis software Image‐Pro Plus.^13^ To avoid that edema could affect the infarct volume value, infarct volume was expressed as the percentage of ischemic damage by dividing the total infarct volume by the total ipsilateral hemispheric volume.^13^ In this way, any potential interference due to increased brain volume caused by water content increase is eliminated.

### 2.11 Evaluation of neurological deficits

Neurological scores were evaluated a few minutes before euthanization according to the following two scales^14^: a general neurological scale and a focal neurological scale. In the general score, the following six general neurological functions were evaluated: (1) hair conditions (score: 0–2), (2) position of ears (score: 0–2), (3) eye conditions (score: 0–4), (4) posture (score: 0–4), (5) spontaneous activity (score: 0–4), and (6) epileptic behavior (score: 0–12). For each of the six general functions measured, animals received a score depending on the severity of the symptoms: the higher the score, the worse is the rat condition. The scores of investigated items were then summed to provide a total general score ranging from 0 to 28. In the focal score, the following seven areas were assessed: (1) body symmetry, (2) gait, (3) climbing, (4) circling behavior, (5) front limb symmetry, (6) compulsory circling, and (7) whisker response. For each of these items, animals were rated between 0 and 4 depending on the severity. The seven items were then summed to give a total focal score ranging between 0 and 28. Infarct volumes and neurological scores were evaluated in a blinded manner by individuals who did not perform the surgical procedures.

### 2.12 Statistical analysis

Values are expressed as means ± SEM. In particular, real‐time PCR results are expressed as *fold change* (2^−ΔΔCt^) compared to the control group set to 1. Briefly, the difference between Ct values of a gene of interest and internal control (ΔCt) is calculated for both the control sample and target sample. Then, the difference between ΔCt of the target sample and control sample (ΔΔCt) is calculated. Fold change of gene expression of target samples compared to control samples is calculated as 2^−ΔΔCt^. For Western blot analysis, results are expressed as percentage of variation of target protein (already normalized for internal control) of the test sample compared to control sample. Statistical analysis was performed with GraphPad Prism 5.0 (GraphPad Software), using ANOVA followed by Newman–Keuls test or Bonferroni post hoc test for more than two groups. To compare two groups, an unpaired *t*‐test was used. Data related to focal and general neurological deficits were analyzed using the non‐parametric test of Kruskal‐Wallis, followed by Dunn's multiple comparison test. Statistical significance was accepted at the 95% confidence level (*p* < 0.05).

## 3 RESULTS

### 3.1 mir135a expression is upregulated 72 h after tMCAO in parallel with the downregulation of its target TRPM7 in the temporoparietal cortex of ischemic rats

**FIGURE 1:**
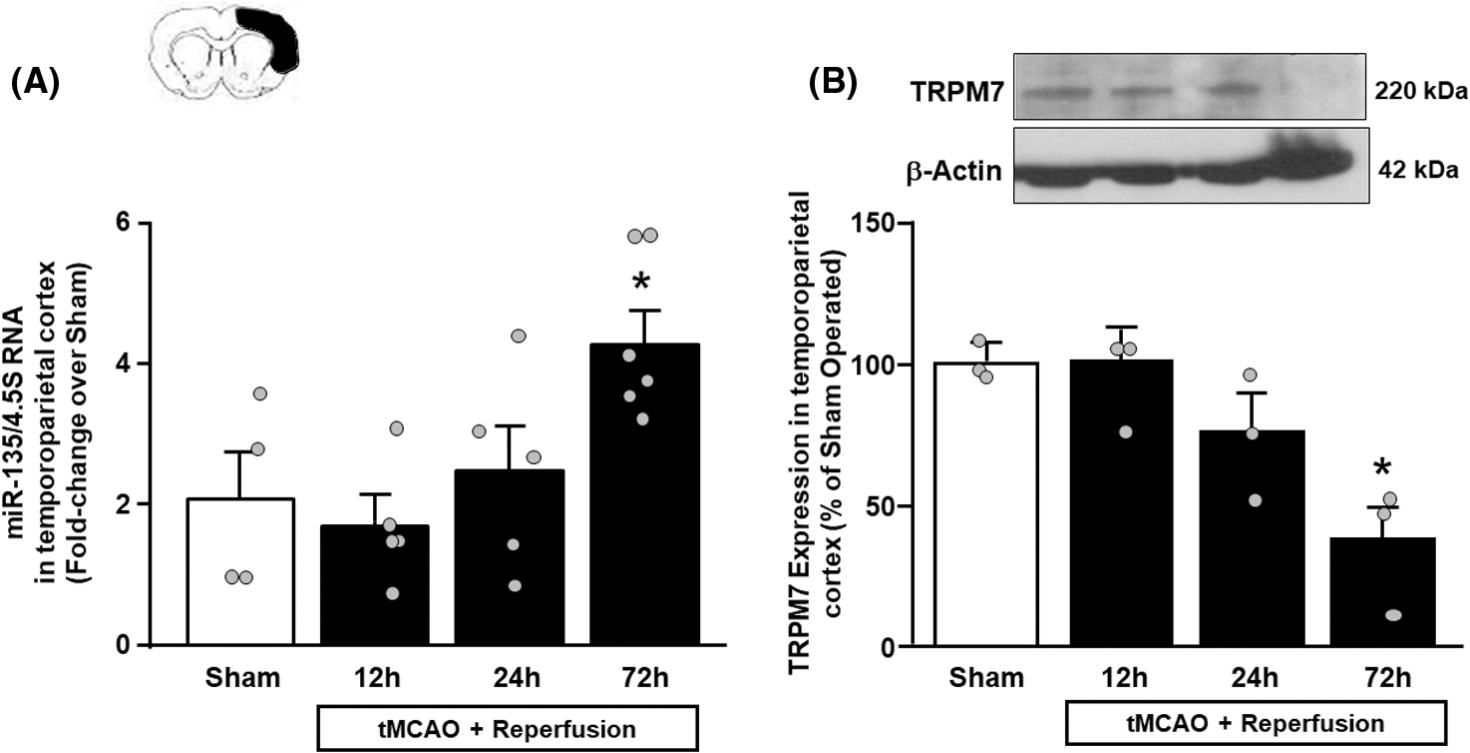
mir135a and TRPM7 expression in the temporoparietal cortex of ischemic rats. Time course of (A) miR‐135 and (B) TRPM7 expression levels in temporoparietal cortex samples harvested from rats subjected to 100′ tMCAO and sacrificed at 12, 24, and 72 h. *p = 0.0084 (A) *p* = 0.010 (B) versus non‐ischemic control rats. Data are expressed as mean ± SEM. Scatter plots are superimposed.

**FIGURE 2:**
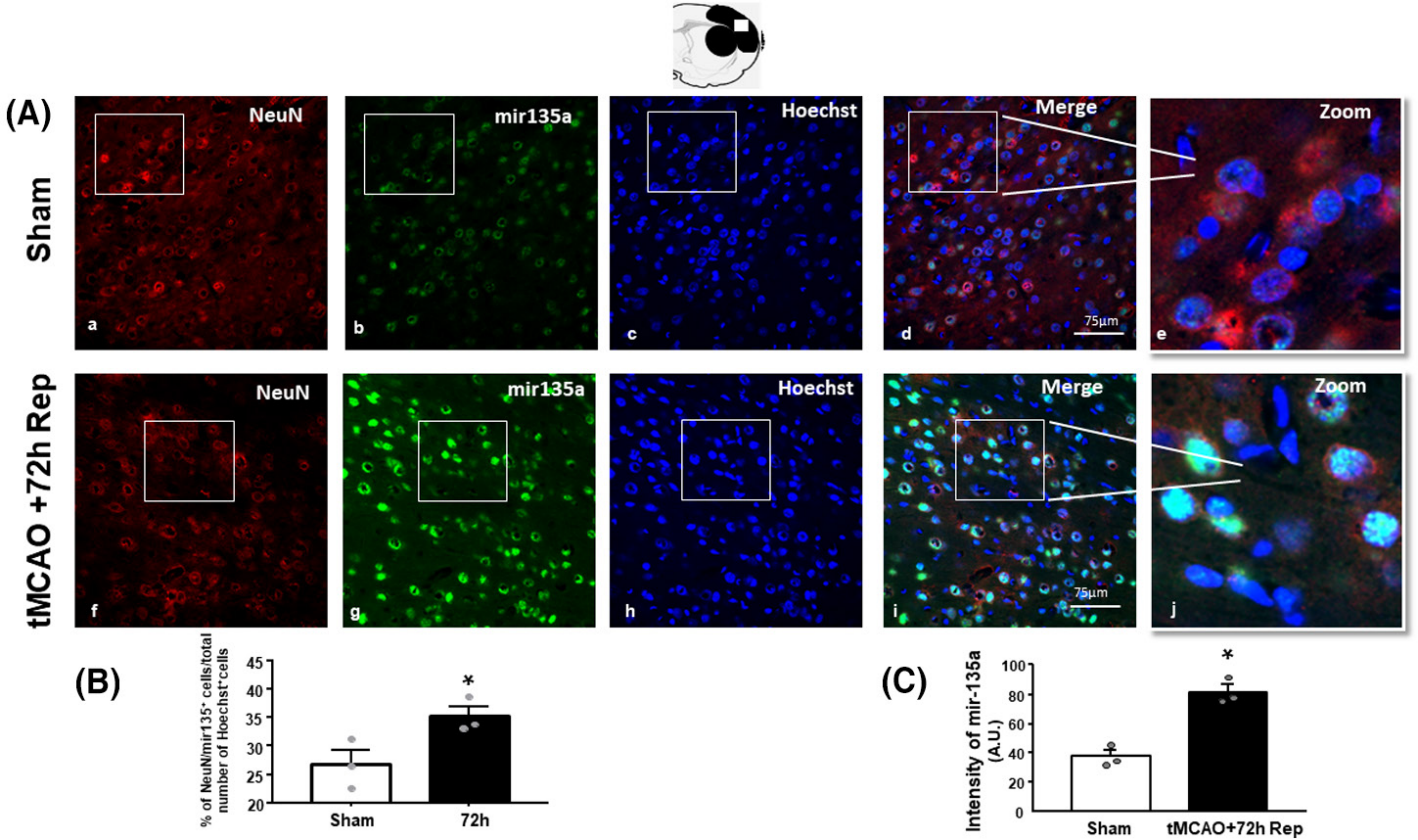
mir135a expression in the temporoparietal cortex of ischemic rats. (A) Confocal microscopic images displaying NeuN in red (a, f), miR‐135a in green (b, g), Hoechst in blue (c, h) and merge in yellow (d, i) in the brain ischemic regions of sham‐operated rats and rats subjected to 100′ tMCAO sacrificed at 72 h. High magnification zoom 2× in pictures (e, j). Scale bars, 75 μm. A representative brain slice cartoon indicating the area of interest is on the top of the figure. Bottom: Quantification of miR‐135a immunoreactivity in neurons. (B) The colocalization was performed by counting the number of NeuN/mir135 positive cells and normalized to the total number of Hoechst positive cells per slide. (C) Image analysis of NeuN was performed by NIH imaging software by measuring the intensity of fluorescent miR‐135a immunolabeling in 120 NeuN‐positive neurons for each group. The intensity of miR‐135a immunoreactivity was expressed in arbitrary units. *n* = 3–4 animals per group. *p = 0.0027 (B) and *p = 0.0497 (C) versus sham‐operated group. Data are expressed as mean ± SEM.

Analysis by RT‐PCR revealed a significant increase in *miR135a* levels in the ipsilateral temporoparietal cortex obtained from ischemic rats at different time intervals from ischemia induction, as compared to non‐ischemic control animals. In particular, a 2‐fold increase was observed at the latest time point, i.e., 72 h, after ischemia (Figure 1A). In parallel with the significant increase of miR‐135a in the cortex, the analysis by Western blot showed a significant decrease in TRPM7 protein levels at the same point, 72 h after tMCAO (Figure 1B). In accordance with RT‐PCR experiments, *miR135a* immuno‐signal determined by FISH analysis increased in the ipsilateral temporoparietal cortex of rats at 72 h after ischemia (Figure 2A). Interestingly, FISH experiments revealed that the localization of *miR135a* was prominent in neurons, in fact the fluorescence intensity of *miR135a* in positive NeuN cells increased compared to sham‐operated animals (Figure 2B). Furthermore, the number of double‐stained cell numbers of NeuN/miR135a^+^ cells significantly increased in ischemic animals compared to sham‐operated animals (Figure 2C).

### 3.2 miR‐135a improves primary cortical neuron survival after OGD + Reoxy and downregulates TRPM7 levels

**FIGURE 3:**
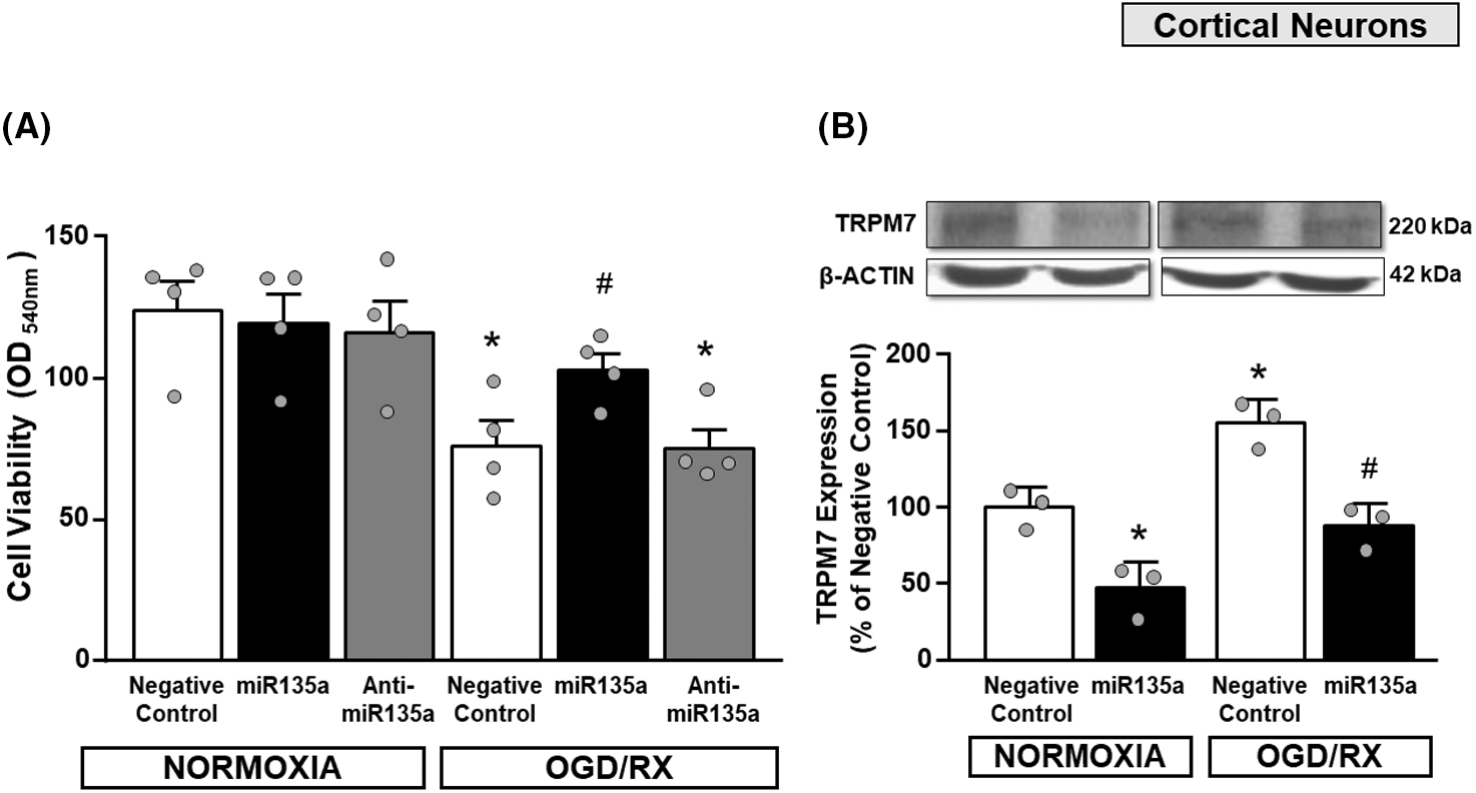
Evaluation of primary cortical neurons survival transfected with mir135 mimic and exposed to OGD + Reoxy. (A) MTT assay on cortical neurons exposed to normoxia or to OGD + Reoxy and incubated with negative control, miR mimic, or the corresponding antimir. ^#^ *p* < 0.05 versus neurons transfected with negative control or antimir and exposed to OGD + Reoxy *p < 0.05 versus neurons transfected with negative control, miRNA or antimir and exposed to normoxia. (B) TRPM7 expression by Western blot analysis in cortical neurons exposed to normoxia and OGD + Reoxy ^#^ *p* = 0.0026 versus neurons transfected with negative control exposed to OGD + Reoxy *p = 0.0107 miRNA + normoxia versus negative control + normoxia, *p = 0.0085 negative control + OGD + Reoxy versus negative control + normoxia. *n* = 3. Each column represents the mean ± SEM.

In order to evaluate the effect of miR135a on cell survival, MTT assay was performed on cortical neurons exposed to normoxia or to Oxygen and Glucose Deprivation followed by Reoxygenation (OGD + Reoxy) and transfected with negative control, miR135a mimic or the corresponding anti‐miR. As shown in Figure 2A, while in normoxic conditions neither the mimic nor the anti‐miR influenced neuronal survival, after OGD + Reoxy neuronal survival significantly decreased in the presence of the negative control or the antimiR135a. On the other hand, transfection of cortical neurons with the mimic miR135a restored neuronal survival to the levels observed during normoxia (Figure 3A).

Interestingly, TRPM7 protein levels were significantly downregulated in neuronal cortical cells transfected with mimic miR135a, both in normoxic and anoxic conditions (Figure 3B).

### 3.3 miR135a mimic ameliorates ischemic damage and improves neurological deficits by inducing TRPM7 downregulation 24 h after ischemia induction

**FIGURE 4:**
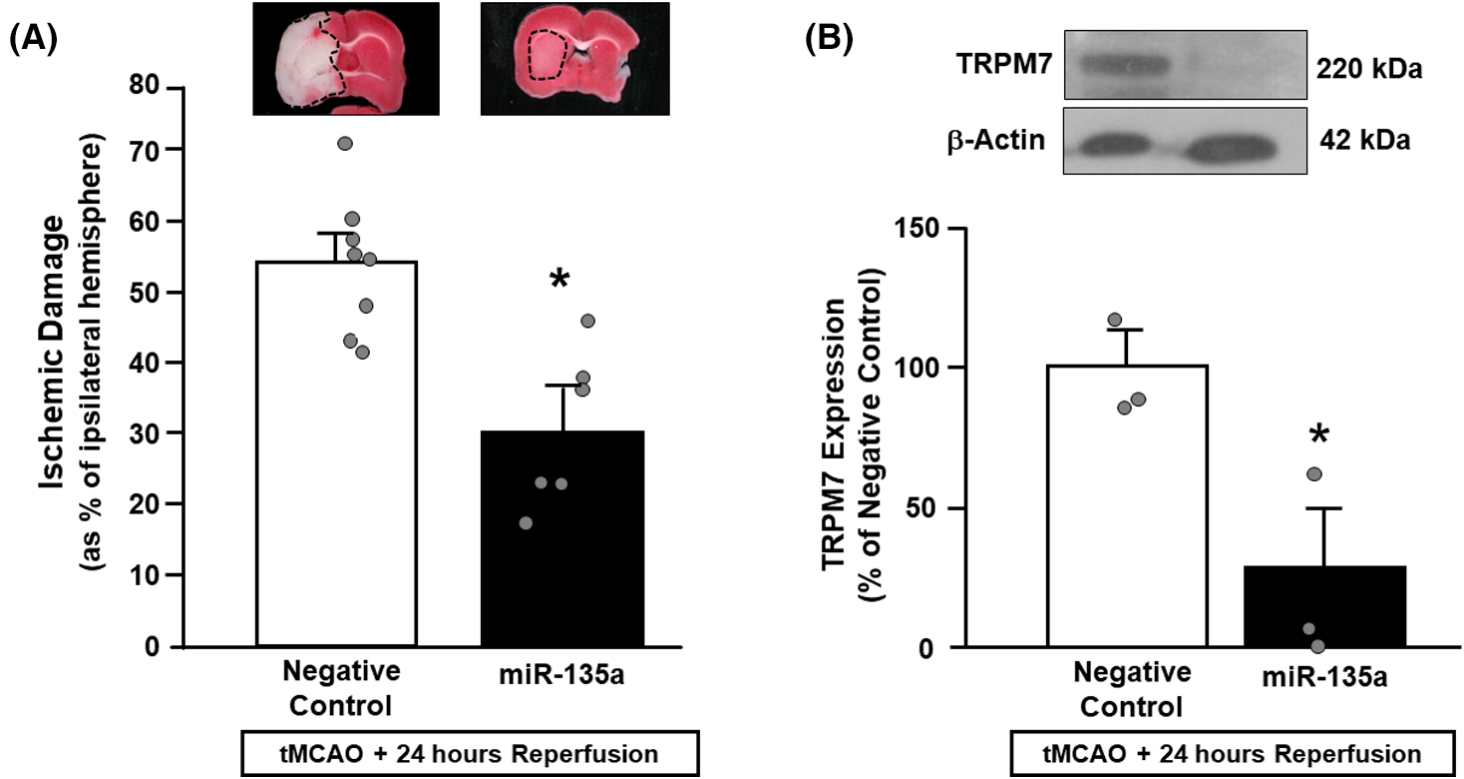
Evaluation of ischemic damage and TRPM7 expression 24 h after tMCAO in rats treated with miR‐135a mimic. (A) Evaluation of ischemic damage 24 h after tMCAO in rats icv perfused with miR‐135a mimic *p = 0.0029 versus ischemic rats treated with negative control *n* = 5–7 animals per group (B) Evaluation of TRPM7 expression 24 h after tMCAO in rats treated with miR‐135a mimic. *p = 0.047 versus ischemic rats treated with negative control, *n* = 3 Each column represents the mean ± SEM. Scatter plots are superimposed.

**FIGURE 5:**
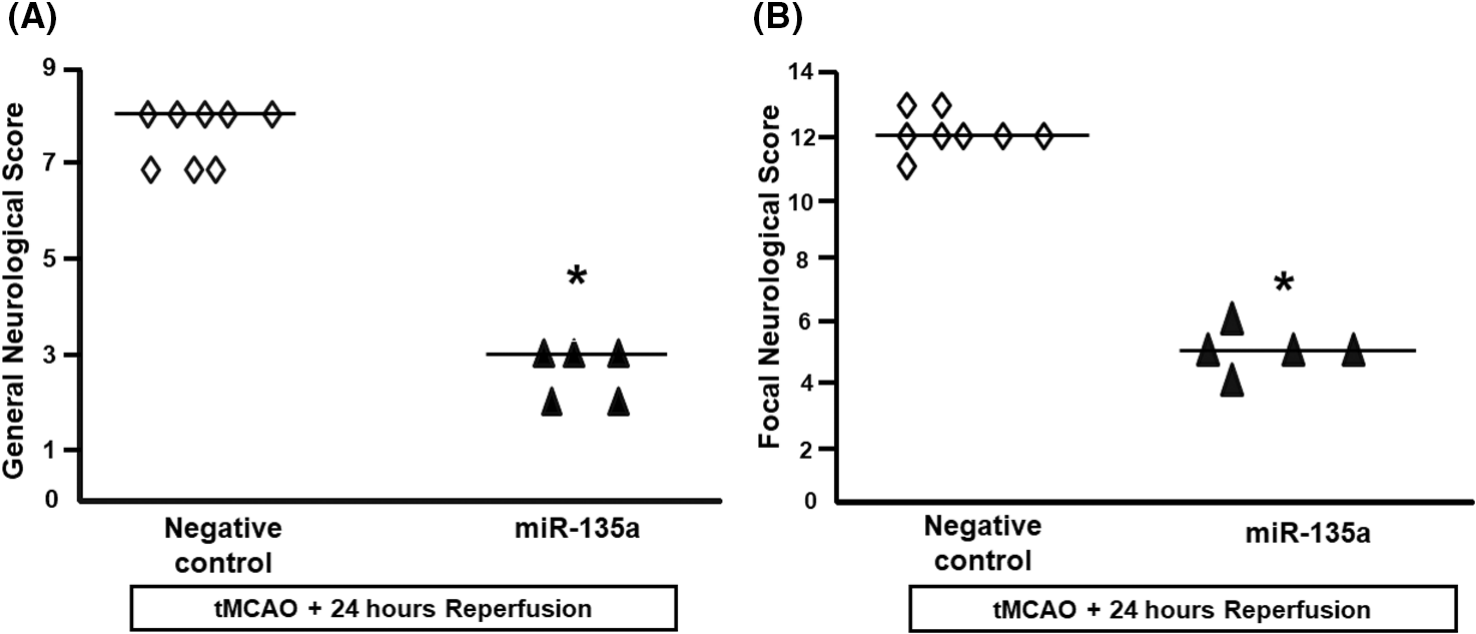
Evaluation of neurological deficits 24 h after tMCAO in rats treated with miR‐135a mimic. General (A) and focal (B) neurological deficits evaluated 24 h after tMCAO in rats treated with miR135a mimic. Neurological scores were analyzed using the non‐parametric Kruskal‐Wallis test, followed by Dunn's multiple comparison test. Statistical significance was accepted at the 95% confidence level, *p < 0.0016 (general score) and 0.0008 (focal score) versus ischemic rats treated with negative control; *n* = 5–7.

To evaluate the effects of miR135a mimic on the extent of the ischemic brain damage, miR135a mimic was icv perfused by an osmotic pump, starting 6 h before ischemia induction and up to 24 h later, as described in the method section. The analysis of the ischemic volume by TTC staining showed a significant reduction (50%) in the ischemic volume of animals treated with miR135a compared to the animals treated with the corresponding negative control (Figure 4A). In order to verify whether the reduction in the ischemic volume was associated with a variation of TRPM7 expression levels, its expression was analyzed by Western blot in the temporoparietal cortex of ischemic rats treated with miR135a mimic and corresponding negative control. Our results showed that miR135a mimic strongly decreased TRPM7 protein level expression in rat temporoparietal cortex 24 h after tMCAO, compared to animals treated with corresponding negative control (Figure 4B). Interestingly, the decrease in ischemic volume and in TRPM7 expression was also accompanied by a parallel improvement of both general and focal neurological deficits (Figure 5A,B). By contrast, post‐stroke miR135a mimic administration was not able to confer neuroprotection (Figure S2).

## 4 DISCUSSION

The results shown in the present paper demonstrated for the first time that miR135a exerts a neuroprotective role in an animal model of focal cerebral ischemia, since its administration strongly ameliorates the ischemic damage and improves neurological function and, more interestingly, this beneficial action is accompanied by a downregulation of TRPM7 protein levels.

Interestingly, we demonstrated that TRPM7 expression is reduced 72 h after ischemia in the ipsilateral temporoparietal cortex and that this decrease parallels the upregulation of miR135a in the same area at the same time interval.

Transient receptor potential melastatin 7 is a Ca^2+^ permeable, non‐selective cation channel that has recently gained attention as a potential cation influx pathway involved in ischemic events. Its role has been extensively clarified in both in vitro and in in vivo models of ischemia. In particular, downregulation of TRPM7 by using siRNA strategy in primary cortical neurons exposed to OGD inhibited ROS‐mediated activation and reduced subsequent cell death.^4^

Recently, Sun and colleagues demonstrated that suppression of TRPM7 channels in vivo by a virally mediated gene silencing through shRNA reduced the death of hippocampal CA1 pyramidal neurons and preserved functions after global cerebral ischemia.^5^ All these findings strongly support the fundamental role of TRPM7 during cerebral ischemia and the medical need of tools able to suppress its activation in order to achieve neuroprotection. Indeed, during the first initial phase of an ischemic attack, hyperactivation of glutamate receptors leads to a large influx of Ca^2+^, which in turn mediates production of nitric oxide (NO) by neuronal nitric oxide synthase (NOS) and production of superoxide (O^2−^) from mitochondria.^15^, ^16^ Along with other factors, such as decreases in pH and extracellular divalent ions, that are associated with ischemic episodes, ONOO^−^ may further enhance TRPM7 activation, which may further impair calcium overload and free radical production. Interestingly, it has been demonstrated that miR‐135a exerts a protective role via promotion of proliferation and suppression of apoptosis and neuroinflammation by targeting GSK3β in MPP^+^‐intoxicated SH‐SY5Y cells, providing a potential therapeutic target for the treatment of Parkinson disease.^17^ The relevance of targeting TRPM7 is also confirmed by the failure of anti‐excitotoxic therapy, which may delay the neurotoxic process but is insufficient to ultimately prevent the subsequent lethal TRPM7 activation. The fact that in our model TRPM7 levels decreased at a late time point after the ischemic damage, i.e., 3 days, may be considered a compensatory reaction of the still savable cortical neurons to counteract the spreading of the ischemic damage. This downregulation completely reflects miR135a upregulation. This hypothesis may support the idea that reducing TRPM7 levels in an early phase after ischemia onset, before the damage has been established, may confer neuroprotection. According to this hypothesis, in the present paper miRNA mimic perfusion has been started 6 h before tMCAO, in order to obtain an earlier miR135a expression, thus consequently causing an earlier reduction in TRPM7 expression, as verified 24 h after ischemia onset. The precocious TRPM7 downregulation was in turn able to effectively reduce the extension of ischemic volume and also to improve neurological functions measured 24 h after reperfusion. This hypothesis is further supported by data on the ineffectiveness of miRNA135 administered to ischemic rats, 3 h after reperfusion.

The presence of an interaction between miR135a and the 3′ UTR of TRPM7, evaluated in the present study by a bioinformatic approach, as shown in Figure S1, has already been validated by a luciferase assay, showing a reduction in luciferase activity when the construct containing the 3′ UTR of TRPM7 was transfected with the miR135a mimic.^6^

Interestingly, our experiments showed that administration of the mimic ameliorates brain ischemia and improves both general and focal neurological deficits by mediating an early reduction in TRPM7 protein levels in the cortex. This finding supports the idea that TRPM7 plays a key role in ionic regulation in this area during ischemic conditions and that the induction of an early downregulation of its expression may help neurons to survive the ischemic injury.

In this context, given the absence of molecules able to selectively inhibit TRPM7 expression or function, the miR mimic strategy may represent a valid and translatable opportunity to modulate the expression of the target protein, considering that miRNAs may be easily encapsulated and conveyed in the brain by nanoparticles. Alternatively, it cannot be excluded that the neuroprotective effect in stroke of miR135a may be attributable also to other targets, different from TRPM7.

Collectively, the results of the present work showed for the first time that the miR135a administration may pave the way for an innovative therapeutic intervention based on an early prevention of TRPM7 activation during brain ischemia.

## AUTHOR CONTRIBUTIONS

Conceptualization: Cuomo, Pignataro; Methodology: Cuomo, Brancaccio, Sirabella, Laudati, Guida, Viscardi; Formal analysis and investigation: Cuomo, Cepparulo, Valsecchi, D'Esposito, Vinciguerra; Formisano Writing – original draft preparation: Cuomo, Pignataro, Annunziato; Funding acquisition: Cuomo, Pignataro, Annunziato; Supervision: Cuomo, Pignataro, Annunziato.

## FUNDING INFORMATION

This work was supported by grants from Fondi FRA 2020 LINEA A EXOREMOTE – CUOMO, to OC; Programma Operativo Nazionale PON PERMEDNET (ArSO1_1226) from the Italian Ministry of Research, MIUR, to LA; PON NEON (ARS01_00769) from the Italian Ministry of Research, MIUR, to GP, PNRR, Neuroscience, Neuropharmacology, “Mnesys”, Spoke 7, to L.A.

## CONFLICT OF INTEREST STATEMENT

The authors declare no conflicts of interest.

## Supporting information


Figure S1

Figure S2

## Data Availability

The data that support the findings of this study are available on request from the corresponding author.
