# Supplementary figures and images for: miR135a administration ameliorates brain ischemic damage by preventing TRPM7 activation during brain ischemia
Source: CNS Neurosci Ther. 2023 Sep 18;30(3):e14448. doi: 10.1111/cns.14448 (PMC10916440; doi:10.1111/cns.14448)

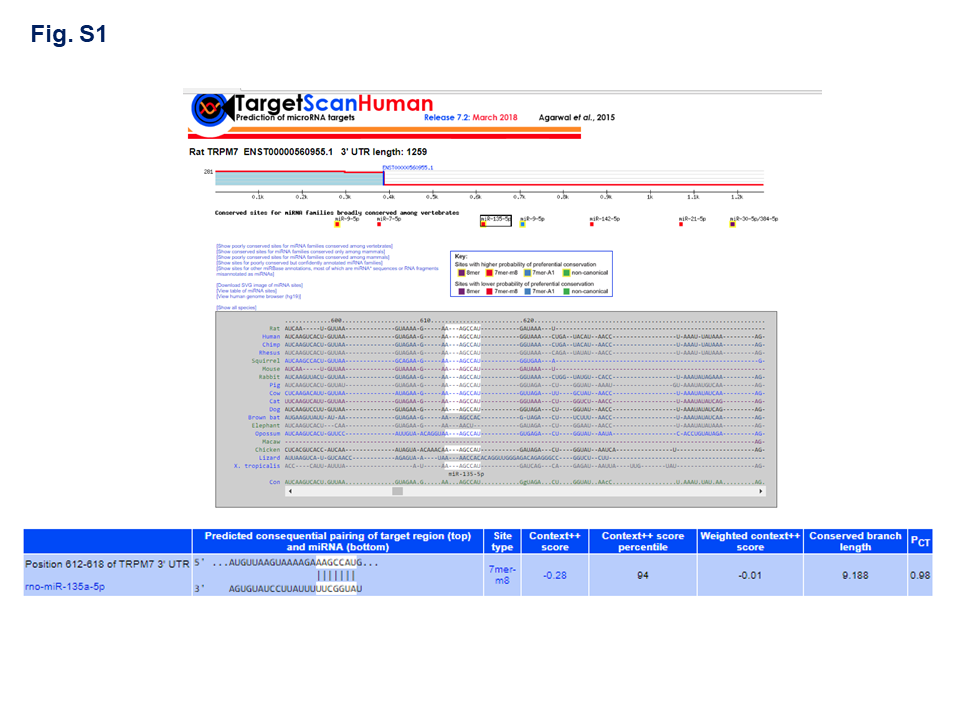

Supplement: Supplementary file 1 — Figure S1 [file CNS-30-e14448-s002.tif]

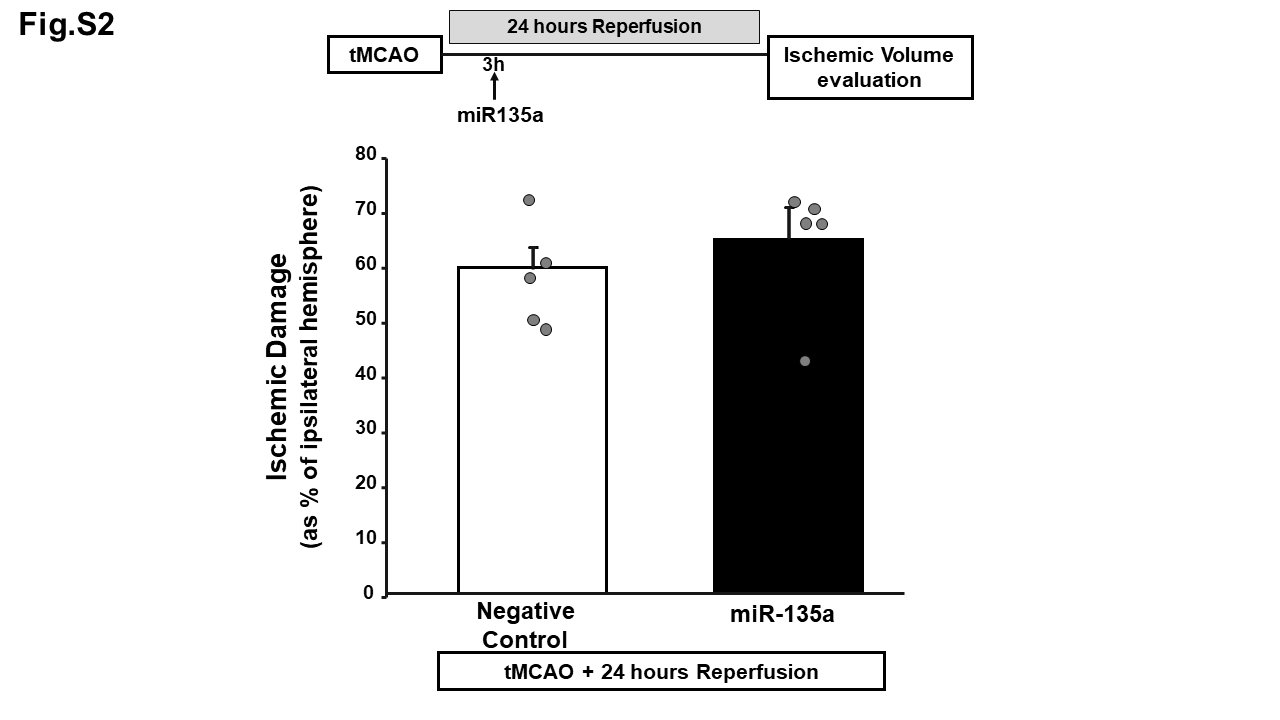

Supplement: Supplementary file 2 — Figure S2 [file CNS-30-e14448-s001.tif]
